# Supplementary material for: Effects of Dapagliflozin on Volume Status When Added to Renin–Angiotensin System Inhibitors
Source: J Clin Med. 2019 May 31;8(6):779. doi: 10.3390/jcm8060779 (PMC6616433; doi:10.3390/jcm8060779)
Supplement: Supplementary file 1 [file jcm-08-00779-s001.pdf]

Supplementary Materials

Figure S1: Design of the IMPROVE study and the DapKid study.

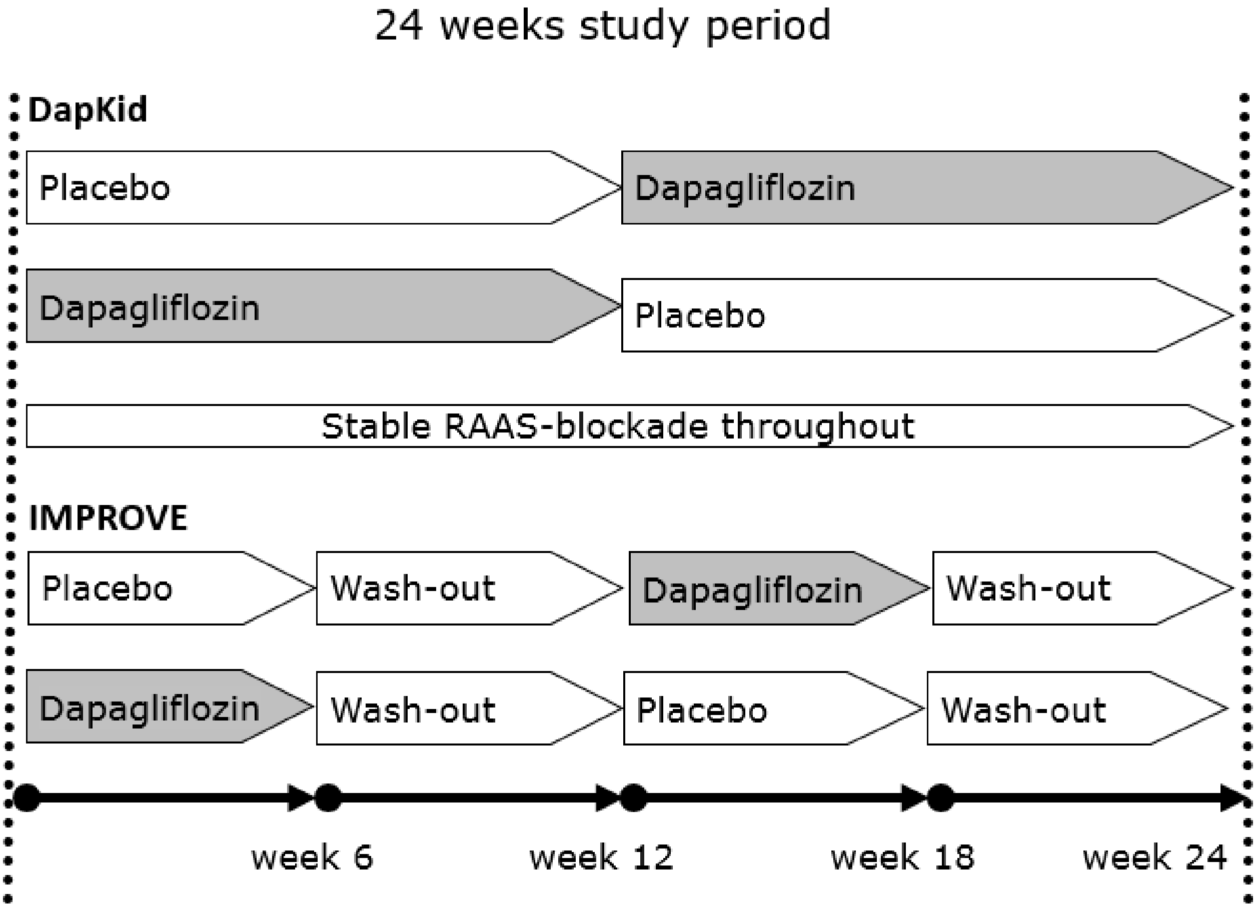

Table S1: Baseline characteristics in the IMPROVE study and DapKid study separately.

Characteristics	IMPROVE (N=33)	DapKid (N=36)
Age (years)	61.4 (9.2)	63.9 (8.3)
Male sex (%)	25 (75.8%)	32 (88.9%)
Diuretic use (%)	26 (78.8%)	17 (50.0%)
Weight (kg)	95.5 (22.3)	103.2 (20.1)
Body mass index (kg/m^2)	31.0 (5.6)	32.8 (5.7)
Systolic blood pressure (mmHg)	141.8 (14.8)	140.7 (15.8)
Diastolic blood pressure (mmHg)	77.3 (5.9)	82.1 (10.0)
Fasting plasma glucose (mmol/L)	8.6 (3.1)	11.0 (3.6)
HbA_{1c} (mmol/mol)	56.8 (9.6)	73.3 (14.8)
Sodium (mmol/L)	139.9 (3.1)	138.6 (2.2)
Potassium (mmol/L)	4.4 (0.4)	4.3 (0.5)
Urea (mmol/L)	6.6 (2.1)	6.1 (2.2)
Osmolality (mOsmol/kg)	287.9 (6.4)	300.6 (16.6)
Copeptin (pmol/L) ‡	9.0 [5.4, 11.2]	9.0 [5.9, 12.6]
Renin (ng/l) ‡	20.6 [10.5, 52.3]	48.0 [24.5, 106.0]
NT-proBNP (ng/l) ‡	68.9 [30.0, 208.9]	110.5 [42.5, 201.0]
Estimated GFR ($mL/min/1.73m^2$)	74.4 (18.3)	84.0 (19.3)
UACR (mg/g)‡	255.1 [114.0, 598.3]	153.8 [91.3, 337.5]
Urinary volume (mL/24h)	1892 (706)	2207 (789)
Urine glucose (mmol/24h)	2.2 [0.7, 14.0]	82.0 [23.0, 372]
Urinary osmolality (mOsmol/kg)	533.9 (163.5)	583.7 (187.6)
Urinary sodium excretion (mmol/24h)	187.8 (82.0)	211.1 (130.7)
Fractional sodium excretion (%)	964.1 (343.3)	932.3 (356.2)
Fractional lithium excretion (%)	11318.7 [88984.9, 17344.4]	Not available
Free water clearance (FWC) (ml/24h)	-1418.7 (858.4)	-1981.0 (1595.7)

Data are given as mean (SD) and ‡median [25th to 75th percentile].

Table S2: Changes in characteristics and volume markers during dapagliflozin treatment versus placebo in the IMPROVE study and DapKid study separately.

Volume markers	Change during dapagliflozin vs placebo, IMPROVE (N=33) (95% CI)	p-value	Change during dapagliflozin vs placebo, DapKid (N=36) (95% CI)	p-value
Weight (kg)	-0.7 (-1.4, -0.1)	p=0.03	-1.9 (-2.6, -1.3)	p<0.01
Systolic blood pressure (mmHg)	-7.7 (-13.2, -2.3)	p<0.01	-3.7 (-8.0, 0.6)	p=0.09
Diastolic blood pressure (mmHg)	-3.0 (-5.4, -0.6)	p=0.02	0.5 (-1.9, 3.0)	p=0.6
Fasting plasma glucose (mmol/L)	-1.0 (-2.2, 0.3)	p=0.11	-2.6 (-3.8, -1.3)	p<0.01
HbA_{1c} (mmol/mol)	-3.1 (-5.7, -0.5)	p=0.02	-7.3 (-10.3, -4.3)	p<0.01
Sodium (mmol/L)	0.3 (-0.5, 1.2)	p=0.3	1.5 (0.7, 2.2)	p<0.01
Potassium (mmol/L)	0.03 (-0.1, 0.2)	p=0.69	-0.07 (-0.2, 0.0)	p=0.18
Urea (mmol/L)	0.9 (0.3, 1.5)	p<0.01	0.07 (-0.5, 0.6)	p=0.8
Osmolality (mOsmol/kg)	1.2 (-0.8, 3.3)	p=0.2	-0.2 (-3.7, 3.3)	p=0.9
Copeptin (%)	33.1% (21.8, 45.5)	p<0.01	33.1% (19.1, 48.9)	p<0.01
Renin (%)	40.3% (11.9, 75.9)	p<0.01	53.5% (13.1, 108.3)	p<0.01
NT-proBNP (%)	5.5% (-14.0, 27.0)	p=0.6	-15.7% (-39.3, 4.1)	p=0.12
Estimated GFR (mL/min/$1.73m^2$)	-6.6 (-9.3, -3.9)	p<0.01	-1.6 (-3.9, 0.7)	p=0.2
Urinary albumin excretion (%)	-57.4 (-89.8, -30.5)	p<0.01	-46.7 (-74.7, -23.2)	p<0.01
Urinary volume (mL/24h)	-1.2 (-135, 133)	p=0.98	534 (233, 835)	p<0.01
Urinary glucose excretion (mmol/24h)	113.7 (108.0, 160.8)	p<0.01	320.5 (194.4, 446.5)	p<0.01
Urinary osmolality (mOsmol/kg)	46.8 (9.5, 84.0)	p=0.02	74.4 (25.5, 123.3)	p<0.01
Urinary sodium excretion (mmol/24h)	-27.7 (-40.6, -14.7)	p<0.01	18.5 (-24.8, 61.8)	p=0.39
Fractional sodium excretion (%)	51.7 (-29.3, 132.7)	p=0.20	153.5 (3.7, 303.4)	p=0.04
Fractional lithium excretion (%)	19.6% (6.7, 34.2)	p<0.01	Not available	
Free water clearance (FWC) (ml/24h)	-444.1 (-702.8, -185.4)	p<0.01	-1330.4 (-1806.9, -853.9)	p<0.01
